# A New Theory of Chloroform Syncope

**Published:** 1890-09

**Authors:** 


*A New Theory of Chloroform Syncope.* By ROBERT KIRK, M.D. Edin. Glasgow: JOHN THOMLINSON. 1890.

The author says in the preface: "Nothing but a strong conviction of being in the right could justify the author of the following pages for expressing views which must give a violent wrench to preconceived opinions, and which will almost certainly be met with opposition from all parties. . . . In justice to himself, and as some explanation of the revolutionary statements here made, it may be added that he believes very few men living have devoted more time and study to the subject than he has done."

We give the new theory in the author's own words:

"The new theory takes its starting-point from the hypothesis that there must be an effect produced by the vapour on the pulmonary mucous membrane, and that we have this also to reckon with. . . . If there be this action on the pulmonary mucous membrane, the agent must be regarded as two forces acting at two several points, one without and the other within the circulation; and the resulting phenomena are to be ascribed to the action and reaction of these two forces, and to the varying degrees of intensity with which they operate at different stages of the inhalation."

The author considers that the action of chloroform upon the circulation may be likened to the effect of a tourniquet upon a large artery, and that its sudden withdrawal will be attended with "the same liability to syncope as would be the sudden removal of the tourniquet."

The practical outcome of the new theory is, that the author recommends the following method of administering chloroform: He requires the apartment to be heated to 65° or 70° F., and has two towels thoroughly warmed and dried at the fire. Two drachms of the liquid are poured on one of the towels, which is then held closely to the nose and mouth; and this dose is repeated in little more than a minute, and always in less than two minutes. Each successive dose is poured on a different part of the towel, not cooled by previous evaporation, which is then reversed so suddenly that the patient never gets a breath of air unimpregnated with the proper percentage of the anæsthetic. He watches the bottle and the towel, and pays nothing but an incidental attention to the pulse or respiration, satisfied that nothing can go wrong with these, short of the deepest surgical anæsthesia.

We cannot say that the author has thrown any new light upon the difficult problems of chloroform anæsthesia, and we are afraid that his method of administration will no more prevent accidents from occasionally happening than any other method.

Our own opinion is in accordance with the views so well stated by Dr. Wood of Philadelphia, in his paper delivered before the recent Berlin Congress:

1. That the use of any anæsthetic is attended with an appreciable risk, and that no care will prevent an occasional loss of life.
2. That chloroform acts much more promptly and much more powerfully than ether, both upon the respiratory centres and the heart.
3. That the action of chloroform is much more persistent and permanent than that of ether.
4. That chloroform is capable of causing death, either by primarily arresting the respiration, or by primarily stopping the heart; but that commonly both respiration and cardiac functions are abolished at or about the same time.

